# Catechin Bioavailability Following Consumption of a Green Tea Extract Confection Is Reduced in Obese Persons without Affecting Gut Microbial-Derived Valerolactones

**DOI:** 10.3390/antiox11122490

**Published:** 2022-12-18

**Authors:** Geoffrey Y. Sasaki, Yael Vodovotz, Zhongtang Yu, Richard S. Bruno

**Affiliations:** 1 Human Nutrition Program, The Ohio State University, Columbus, OH 43210, USA; 2 Department of Food Science and Technology, The Ohio State University, Columbus, OH 43210, USA; 3 Department of Animal Sciences, The Ohio State University, Columbus, OH 43210, USA

**Keywords:** green tea, catechins, antioxidant, gut microbiota, valerolactones, pharmacokinetics

## Abstract

Obesity-related cardiometabolic disorders are driven by inflammation, oxidative stress, and gut dysbiosis. Green tea catechins protect against cardiometabolic disorders by anti-inflammatory, antioxidant, and prebiotic activities. However, whether obesity alters catechin bioavailability remains unknown. We hypothesized that obesity would decrease catechin bioavailability due to altered gut microbiota composition. Obese and healthy persons completed a pharmacokinetics trial in which a confection formulated with green tea extract (GTE; 58% epigallocatechin gallate, 17% epigallocatechin, 8% epicatechin, 6% epicatechin gallate) was ingested before collecting plasma and urine at timed intervals for up to 24 h. Stool samples were collected prior to confection ingestion. Catechins and γ-valerolactones were assessed by LC-MS. Obesity reduced plasma area under the curve (AUC_0-12h_) by 24–27% and maximum plasma concentrations by 18–36% for all catechins. Plasma AUC_0-12h_ for 5′-(3′,4′-dihydroxyphenyl)-γ-valerolactone and 5′-(3′,4′,5′-trihydroxyphenyl)-γ-valerolactone, as well as total urinary elimination of all catechins and valerolactones, were unaffected. ⍺-Diversity in obese persons was lower, while *Slackia* was the only catechin-metabolizing bacteria that was altered by obesity. Ascorbic acid and diversity metrics were correlated with catechin/valerolactone bioavailability. These findings indicate that obesity reduces catechin bioavailability without affecting valerolactone generation, urinary catechin elimination, or substantially altered gut microbiota populations, suggesting a gut-level mechanism that limits catechin absorption.

## 1. Introduction

Obesity is a major public health issue with over two-thirds of Americans classified as overweight or obese [1]. Low-grade chronic inflammation and oxidative stress accompany obesity [2,3] and are at least partly attributed to gut barrier dysfunction that promotes endotoxemia-associated inflammation [4]. Impairments in gut barrier function due to intestinal inflammation or gut dysbiosis can drive metabolic dysfunction and disrupt host metabolism [5,6]. Obesity-associated metabolic impairments provoke the development of more serious diseases (e.g., cardiovascular disease, diabetes), and may significantly limit the bioavailability, and hence efficacy, of bioactive food components with antioxidant and anti-inflammatory activities that could mitigate disease progression. 

Catechin-rich green tea extract (GTE) exerts antioxidant and anti-inflammatory activities that protect against obesity in rodents by reducing gut permeability that otherwise provokes endotoxemia-mediated inflammation [7,8,9]. Clinical studies support that green tea consumption alleviates dyslipidemia [10] and increases antioxidant biomarkers [11,12]. Health benefits of GTE are attributed to its parental catechins [e.g., epigallocatechin gallate (EGCG), epigallocatechin (EGC), epicatechin gallate (ECG), epicatechin (EC)]. The bioavailability of polyphenolic catechins has been studied extensively in healthy persons, with evidence showing that maximum plasma concentrations reach 0.07–1.8 µM within 1–3 h of ingestion and their half-lives range from 2.5 to 5.7 h [13]. However, these studies in healthy persons fail to consider physiological impairments common in obesity, such as inflammation/oxidative stress, gut barrier dysfunction, and gut dysbiosis, that may alter catechin absorption, distribution, metabolism, and elimination [14,15,16,17]. Indeed, inflammation is proposed to increase paracellular absorption of catechins, limit intestinal and hepatic catechin efflux by limiting phase III transporters, and inhibit phase II xenobiotic enzymes [17]. Furthermore, the bioavailability of parental catechins is poor, with <1% of the ingested dose reaching circulation [18] and ~70% of total catechins accumulating in the distal gut [19].

The well-established poor bioavailability of GTE catechins has resulted in a paradigm shift to focus on the role of microbial-derived metabolites of catechins (e.g., γ-valerolactones; VLs) in disease prevention [20] consistent with their relatively higher absorptive rates and anti-inflammatory activities [21]. However, the bioavailability of γ-VLs has only been studied in limited healthy cohorts [22,23] and none have examined catechin metabolism and bioavailability relative to the gut microbiota. Evidence *in vitro* has identified several gut bacteria capable of metabolizing catechins [24], but studies *in vivo* are scarce [25]. Like catechins, obesity may influence the generation and/or bioavailability of catechin-derived microbial metabolites by altering gut microbiota composition. Since the formation of γ-VLs is microbiota-dependent and obesity is associated with gut dysbiosis [15], obesity could dysregulate microbial biosynthesis and hence bioavailability of γ-VLs. However, no studies have examined the influence of obesity in relation to the gut microbiota and γ-VL bioavailability.

In the present study, we hypothesized that obesity would impair GTE catechin bioavailability in association with decreased microbial-derived γ-VLs resulting from reduced abundance of catechin-metabolizing bacteria. To test this, we conducted a study in obese and lean persons to assess catechin/γ-VL pharmacokinetics parameters and gut microbiota composition. Outcomes of these novel studies are therefore expected to inform the consequences of obesity relative to establishing effective dietary intakes of catechins that can alleviate pathogenic cardiometabolic responses in humans.

## 2. Materials and Methods

### 2.1. Materials

All solvents were LC/MS or HPLC-grade and were purchased from Fisher Scientific (Waltham, MA, USA). EGCG, EGC, ECG, and EC standards were purchased from Sigma-Aldrich (St. Louis, MO, USA). We purchased 5′-(3′,4′-dihydroxyphenyl)-γ-VL (3,4-VL) from BOC Sciences (Shirley, NY, USA). Decaffeinated GTE was from Taiyo International (90LB Sunphenon; Minneapolis, MN, USA). We verified its composition by HPLC-UV [26] to contain 89% *w*/*w* total catechins (64.7% EGCG, 19.4% EGC, 8.7% EC, and 6.2% ECG).

### 2.2. Participants

This protocol was approved by the Institutional Review Board at The Ohio State University (#2017H0246). Obese/overweight and age-matched lean persons were recruited from the Columbus, Ohio area and provided written informed consent prior to enrollment. Obese/overweight participants were required to have a BMI of 28–40 kg/m^2^ and fasting glucose <126 mg/dL, while lean individuals were required to have a BMI of 19–24 kg/m^2^, be normoglycemic (<100 mg/dL), normolipidemic (total cholesterol <240 mg/dL, triglyceride <150 mg/dL), and normotensive (<120/80 mmHg). Additionally, all participants were required to meet the following criteria: 18–50 years, nonsmoker, non-dietary supplements user, non-regular tea drinkers (<2 cups/wk), non-use of any medications or antibiotics, non-pregnant, <3 drinks/d of alcohol, and no pre-existing gastrointestinal disorders/surgeries.

### 2.3. GTE Confection Formulation

Epidemiological evidence suggests that high green tea intakes protect against cardiometabolic disorders [27,28]. As an alternative to high fluid intakes of green tea, especially among Western populations that prefer black tea [29], we developed a novel confection as a delivery vehicle for catechins to assess intestinal, anti-inflammatory activities of GTE catechins in metabolic syndrome patients as described [30]. For the present pharmacokinetics trial, we used this same formula with minor modifications to deliver 0.5 g GTE (290 mg EGCG, 87 mg EGC, 39 mg EC, 28 mg ECG) to each participant. Briefly, a 100 g confection was prepared by heating a mixture that contained 84.5% water, 2% sucrose, 6% gelatin, 0.5% citric acid, 6% lime-flavored gelatin, and 1% decaffeinated GTE powder. The gelatin-based mixture was then molded into a rectangular cube, cooled overnight at 4 °C, and then a 50 g portion containing 500 mg total catechins was provided to each study participant. All confections were prepared 24 h prior to each participants’ scheduled trial and stored at 4 °C in airtight plastic bags to prevent catechin degradation. In-house testing indicated that catechins were stable for at least 7 days under these conditions. 

### 2.4. Study Design

Participants arrived in the morning at the study center in a fasted state (10–12 h) and were asked to void their bladder prior to assessing their height, weight, waist circumference, and blood pressure. Participants then ingested a GTE-containing confection (50 g confection containing 0.5 g GTE) without any additional foods, except water (500 mL). Blood samples were then collected before (0 h, baseline) and after ingestion of the GTE confection at 0.25, 0.5, 1, 2, 3, 5, 8, 10, and 12 h from an in-dwelling catheter that was placed in the antecubital fossa. Additionally, urine samples were collected at timed intervals (0–4, 4–8, 8–12, and 12–24 h) during the trial.

Prior to the pharmacokinetics trial, participants collected one stool sample daily for 3 consecutive days. Three days prior to the pharmacokinetics trial, participants avoided consuming any polyphenol-rich foods/beverages to limit any confounding effects during the trial. Standardized meals devoid of polyphenols were provided to participants in a eucaloric manner based on the Harris–Benedict formula [31] during the 24 h pharmacokinetic trial. Standardized meals provided 48–55% of energy from carbohydrate, 15–20% from protein, and 20–35% from fat. Participants consumed breakfast at 4 h and lunch was provided between 5–8 h during the trial. Participants consumed dinner after their 12 h blood draw and snacks could be consumed any time after lunch and dinner. Only prescribed foods and beverages were consumed by participants during the 24 h trial.

### 2.5. Biospecimen Collection, Handling, and Preservation

Fresh stool samples, obtained on 3 consecutive days within 3–5 days of the trial, were collected into specimen commodes, stored on ice in insulated coolers, and returned to the study center within 24 h of collection. Stool samples for each participant were pooled, aliquoted in sterile cryotubes (~3 g), snap-frozen in liquid nitrogen, and stored at −80 °C until microbiota analysis.

Venous blood was collected into evacuated tubes containing ethylenediaminetetraacetic acid (EDTA) for clinical chemistries and sodium heparin for catechin analysis. Plasma was obtained by immediately centrifuging the samples (3000× *g*, 15 min, 4 °C) upon collection, aliquoting the plasma into cryovials, and snap-freezing in liquid nitrogen. Urine was collected at timed intervals over the 24 h period. Prior to aliquoting the urine, the total volume of urine for each interval was measured and recorded.

To preserve catechins/catechin metabolites in plasma and urine, 100 µL of a preservative solution was added to 1 mL of heparin-treated plasma/urine to obtain a final concentration of 2% (*v*/*v*) acetic acid, 0.02% (*w*/*v*) ascorbic acid, and 0.01% (*w*/*v*) EDTA. Samples were then inverted and snap-frozen in liquid nitrogen. For measures of plasma ascorbic acid and uric acid, 500 µL of heparin-treated plasma was mixed with 500 µL of 10% (*w*/*v*) perchloric acid containing 1 mM diethylenetriaminepentaacetic acid, vortexed, and centrifuged (13,000× *g*, 5 min, 4 °C). The supernatant was then transferred to a cryovial and snap-frozen in liquid nitrogen. All biospecimens were stored at −80 °C until analyzed.

### 2.6. Plasma and Urinary Catechins and γ-VL Analysis

Plasma and urinary catechins (EGCG, EGC, ECG, EC) and γ-VLs [5′-(3′,4′-dihydroxyphenyl)-γ-VL (referred to as 3,4-VL) and 5′-(3′,4′,5′-trihydroxyphenyl)-γ-VL (referred to as 3,4,5-VL)] were extracted following enzymatic hydrolysis according to established methods [32,33] with minor modifications. Briefly, 200 µL of plasma or urine was mixed with 30 µL ethyl gallate (internal standard; 2 µM), 20 µL of ascorbic acid-EDTA solution (20% *w*/*v* ascorbic acid, 0.1% *w*/*v* EDTA in water), 500 µL of sodium acetate buffer (0.1 M, pH 5.0), and 25 µL of sulfatase/β-glucuronidase solution (~40 U sulfatase, ~400 U β-glucuronidase). *Helix pomatia*-derived sulfatase/β-glucuronidase (54.2 mg/mL; Sigma-Aldrich #S9626) was mixed with 0.2% (*w*/*v*) sodium chloride in water to prepare the enzyme solution, aliquoted, and stored at −20 °C. Samples were then gently vortexed and incubated in a shaking water bath at 37 °C for 45 min. Samples were then cooled on ice (~1 min), extracted with 4 mL of ethyl acetate by hand inversion (60 s), and centrifuged (1000× *g*, 6 min, 4 °C). A known volume of the upper layer was transferred to a new glass tube, dried under nitrogen gas in a water bath (35 °C), and then reconstituted in 100 µL of 30% (*v*/*v*) methanol containing 0.1% (*v*/*v*) formic acid. Following centrifugation (15,000× *g*, 10 min, 4 °C), the supernatant was analyzed by LC-MS.

Catechins/γ-VLs were assessed using a Shimadzu LCMS-2020 instrument (Columbia, MD, USA). Samples (2 µL) were separated at 0.2 mL/min at 40 °C using a binary linear gradient on a Waters Acquity UPLC BEH C18 column (100 mm × 2.1 mm, 1.7 µm; Waters Corp, Milford, MA, USA). Mobile phase A consisted of 0.1% formic acid in water (*v*/*v*) and mobile phase B was 0.1% formic acid in methanol (*v*/*v*). The following gradient profile was used: initial conditions of 20% B for 1 min, followed by an increase to 95% B over 6 min, which was held for 1 min, and then a linear decrease to 20% B over 1 min was performed prior to column re-equilibration for 3 min at 20% B. Individual catechins and γ-VLs were measured under negative electrospray ionization in single ion monitoring mode at corresponding mass-to-charge ratios (*m/z*) for each catechin (*m/z* 457, EGCG; *m/z* 441, ECG; *m/z* 305, EGC; *m/z* 289, EC) and γ-VLs (3,4-VL, *m/z* 207; 3,4,5-VL, *m/z* 223). Nebulizing and drying gases were supplied at 1.5 and 15 L/min, and block and desolvation line temperatures were 400 °C and 250 °C, respectively.

Catechin and γ-VL concentrations were calculated from standard curves that were comprised of comparing the ratio for the peak areas of each authentic standard to the area of the internal standard. Because no authentic standard was available for 3,4,5-VL, this metabolite was identified by performing in-source fragmentation studies to monitor precursor and product ion (223→179) based on prior reports [34]. Additionally, 3,4,5-VL was quantified against the 3,4-VL authentic standard. Urinary concentrations of catechins/γ-VLs were normalized to the total urinary volume collected at each collection interval to determine the mass of each compound excreted.

### 2.7. Clinical Chemistries and Plasma Ascorbic Acid and Uric Acid

Plasma glucose, triglyceride, total cholesterol, high-density lipoprotein-cholesterol (HDL-C), and alanine and aspartate aminotransferases were measured using a UV2600 spectrophotometer (Shimadzu; Columbia, MD, USA) using separate clinical assays according to the manufacturer’s instructions (Pointe Scientific; Canton, MI, USA). Insulin was measured by ELISA following the manufacturer’s instructions (Alpco; Salem, NH, USA) using a Synergy H1 microplate reader (Biotek Instruments; Winooski, VT, USA). The homeostatic model assessment of insulin resistance (HOMA-IR) was calculated using fasting plasma glucose and insulin as described [35]. Ascorbic acid, the major water-soluble antioxidant, was assessed due to its known low concentrations in persons with metabolic syndrome [36], which could alter catechin bioavailability. Perchloric acid-treated plasma samples were used to simultaneously measure ascorbic acid and uric acid by HPLC-ECD as we described [37].

### 2.8. Gut Microbiota Composition

Total DNA was extracted from 3-day pooled fecal samples using the QIAamp Fast DNA Stool Mini Kit (Qiagen; Redwood City, CA, USA) as previously described [38]. From the extracted DNA (5 µg), the V4–V5 hypervariable region of the 16S rRNA gene was amplified by PCR to prepare amplicon libraries which were pair-end sequenced (2 × 300) using an Illumina MiSeq sequencer (San Diego, CA, USA). [7]. Paired-end reads were analyzed using QIIME2 (version 2019.10 obtained from http://qiime.org accessed 30 September 2022) [39]. The QIIME2 workflow consisted of initially removing primers and adapters from sequences. Trimmed sequences were then processed by DADA2 to perform sequence denoising, merging of forward and reverse reads, and removing chimeric sequences [40]. Forward and reverse reads were trimmed when the quality score of the reads fell below 25. A rarefied feature table was then used to assess α- (i.e., Chao1, Shannon) and β-diversity (i.e., Bray–Curtis dissimilarity) metrics. Taxonomic analysis was performed by using the reference sequences and taxonomy annotation files from the Silva database (release 132). A Naïve Bayes classifier using the 99% 16S reference sequence data set and raw.taxonomy files from the Silva database were used to create a trained classifier. The trained classifier was then applied to the DADA2 feature table to assign the appropriate taxonomies. 

### 2.9. Statistical Analysis

Plasma pharmacokinetics parameters of catechins and γ-VLs including maximum plasma concentration (C_max_) and time to maximum plasma concentration (T_max_) were assessed in Microsoft Excel. Area under the curve (AUC_0-12h_) was calculated according to the trapezoidal rule using GraphPad Prism (San Diego, CA, USA). Data (means ± SEM) were analyzed using a Student’s independent *t*-test to assess between-group differences of most endpoints. For microbiota, Bray–Curtis dissimilarities were calculated, and individual samples were visualized using principal coordinate analysis and permutational multivariate analysis of variance (PERMANOVA) to assess group differences. Linear regression analysis between study variables was performed to assess pairwise correlations. Data were normally distributed or achieved through log transformation. Statistical significance for all analyses was established at *p* ≤ 0.05.

## 3. Results

### 3.1. Participant Characteristics, Clinical Chemistries, Ascorbic Acid, and Uric Acid

All obese participants (*n* = 10 M/7F) and lean participants (*n* = 10 M/9F) completed the pharmacokinetics trial without any adverse events. As planned, the BMI of obese participants was significantly greater than that of lean participants (Table 1). Obese participants also had significantly greater waist circumferences (Table 1). Systolic and diastolic blood pressure were higher in obese persons but were normotensive (Table 1). They also had higher plasma alanine aminotransferase and insulin, lower ascorbic acid, lower HDL-C and HOMA-IR, but no differences in plasma glucose, triglyceride, total cholesterol, and aspartate aminotransferase; all values were within normal clinical limits.

### 3.2. Obesity Reduces Plasma Bioavailability of Catechins without Affecting Microbial-Derived γ-VLs

**Figure 1:**
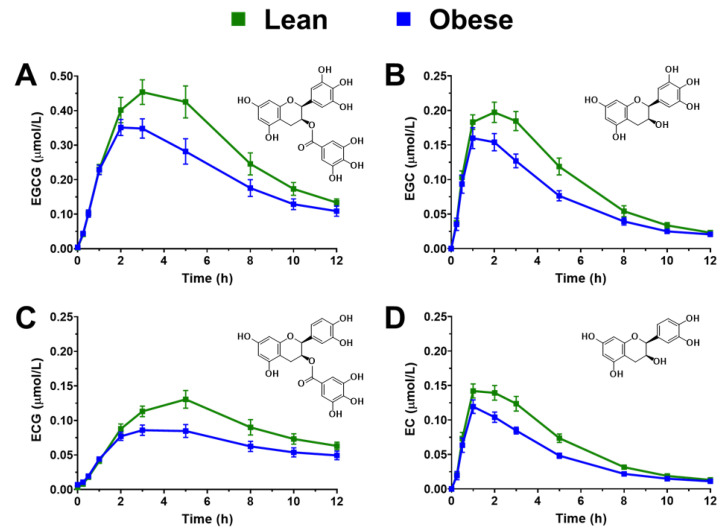
Plasma concentrations (means ± SEM) of (**A**) EGCG, (**B**) EGC, (**C**) EC, and (**D**) ECG in lean and obese persons (*n* = 17–19 per group) after oral ingestion of a GTE-rich confection containing 290 mg EGCG, 87 mg EGC, 39 mg EC, and 28 mg ECG. Abbreviations: EC, epicatechin; ECG, epicatechin gallate; EGC, epigallocatechin; EGCG, epigallocatechin gallate.

Time-dependent plasma concentrations of GTE catechins in obese and lean persons are shown in Figure 1. Obese persons had 24–27% lower plasma AUC_0-12h_ for the four parental catechins relative to lean individuals (*p* < 0.05; Table 2). The CV_Inter-individual_ for the AUC_0-12h_ of each catechin within each group ranged from 29.2–39.3% (Table 2). Consistent with lower AUC_0-12h_, obese persons had 18–36% lower plasma C_max_ for the four parental catechins compared to lean persons. However, terminal (i.e., 12 h) plasma concentrations of each catechin did not differ by health status (Table 2). No significant differences were observed between groups for T_max_ for all catechins (Table 2), but the T_max_ of gallated catechins (EGCG, ECG) was significantly later than that of non-gallated catechins (EGC, EC) regardless of health status (Figure 1A–D). In both obese and lean persons, the C_max_ of each catechin accounted for <1% of the dose of each catechin ingested in agreement with the known poor plasma bioavailability of catechins in humans [18].

**Figure 2:**
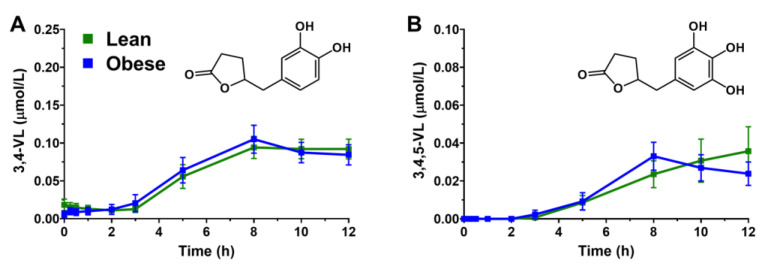
Plasma concentrations (means ± SEM *n* = 17–19 per group) of (**A**) 3,4-VL and (**B**) 3,4,5-VL in lean and obese persons after oral ingestion of a GTE-rich confection containing 290 mg EGCG, 87 mg EGC, 39 mg EC, and 28 mg ECG. Abbreviations: 3,4-VL, 5′-(3′,4′-dihydroxyphenyl)-γ-valerolactone; 3,4,5-VL, 5′-(3′,4′,5′-trihydroxyphenyl)-γ-valerolactone; EC, epicatechin; ECG, epicatechin gallate; EGC, epigallocatechin; EGCG, epigallocatechin gallate.

We hypothesized that decreased catechin bioavailability in obese persons could be attributed to higher production of microbial-derived γ-VLs. Plasma 3,4-VL was detected in all participants, while 3,4,5-VL was detected in 74–76% of lean and obese persons (Table 3). Despite reduced parental catechin AUC_0-12h_ and C_max_ in obese persons, plasma AUC_0-12h_ and C_max_ of both 3,4-VL and 3,4,5-VL were unaffected by obesity status (Table 3). However, the AUC_0-12h_ and C_max_ for 3,4-VL was greater compared to 3,4,5-VL regardless of health status, which is an interesting observation since EGCG and EGC represent 85% of the total catechins in the confection and are the major precursors to 3,4,5-VL (Figure 2A,B). Consistent with prior reports [22,23], plasma 3,4-VL and 3,4,5-VL peaked (8–10 h) in plasma several hours after the T_max_ of their parental catechin counterparts (Table 3). The CV_Inter-individual_ for 3,4-VL and 3,4,5-VL AUC_0-12h_ were quite large (59.0–125.8%), suggesting high within-group variability to generate these gut microbiota-derived metabolites (Table 3). Thus, obesity reduces plasma catechin bioavailability, but without affecting microbial generation of γ-VLs. 

### 3.3. Urinary Elimination of Catechins and γ-VLs were Unaffected by Obesity

We next hypothesized that reduced catechin bioavailability in obese persons could be attributed to more rapid elimination of these compounds. We therefore examined urinary catechins and γ-VLs from complete 24 h urine collections. Obesity status did not alter total 24 h urinary output of any catechins or γ-VL (Table 4). Despite this, there were significant differences in urinary accumulation among the different catechins and γ-VLs. Regardless of health status, non-gallated catechins (EGC, EC) were predominantly found in the urine, whereas little to no gallated catechins (EGCG, ECG) were detected (Table 4). The amount of urinary EGC and EC was ~300–500 times greater than that of EGCG and ECG despite these gallated catechins accounting for 72% of the total catechins ingested (Table 4). Despite this incongruence, the total amount of parental catechins accumulated over 24 h only accounted for 0.01–1.84% of the ingested catechin dose (Table 4). Additionally, total urinary γ-VLs (i.e., sum of 3,4-VL and 3,4,5-VL) were significantly higher in both obese and lean groups relative to the total urinary concentrations of all catechins (Table 4). Notably, 3.3 mg of 3,4-VL on average accumulated in the urine of obese and lean participants, whereas the levels of urinary 3,4,5-VL was ~73% lower (0.9 mg) (Table 4). Additionally, urinary 3,4-VL and 3,4,5-VL output only accounted for a small percentage of the ingested dose of catechins. These findings suggest that obesity does not alter 24 h urinary elimination of parental and γ-VLs, but non-gallated catechins and γ-VLs are predominantly eliminated in the urine compared to gallated catechins.

### 3.4. Obesity Lowers Gut Microbiota α-Diversity without Significantly Affecting Microbial Populations Associated with Catechins and Xenobiotic Metabolism

**Figure 3:**
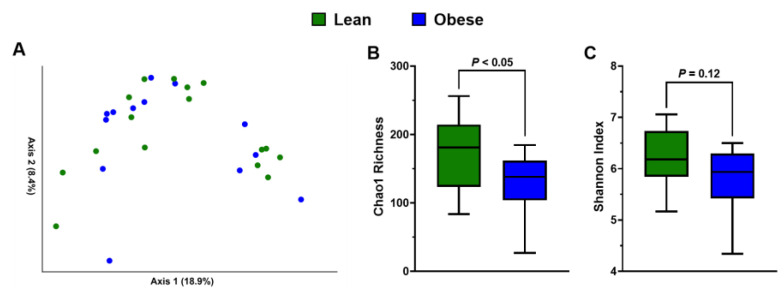
α- and β-Diversity measures of gut microbiota in obese and lean persons. (**A**) Principal coordinate analysis of Bray–Curtis dissimilarity of gut microbiota, (**B**) Chao1 richness estimate, and (**C**) Shannon index of obese (*n* = 13) and lean (*n* = 16) persons prior to the pharmacokinetics trial. Data were analyzed using QIIME2 (version 2019.10 obtained from http://qiime.org accessed 30 September 2022).

**Figure 4:**
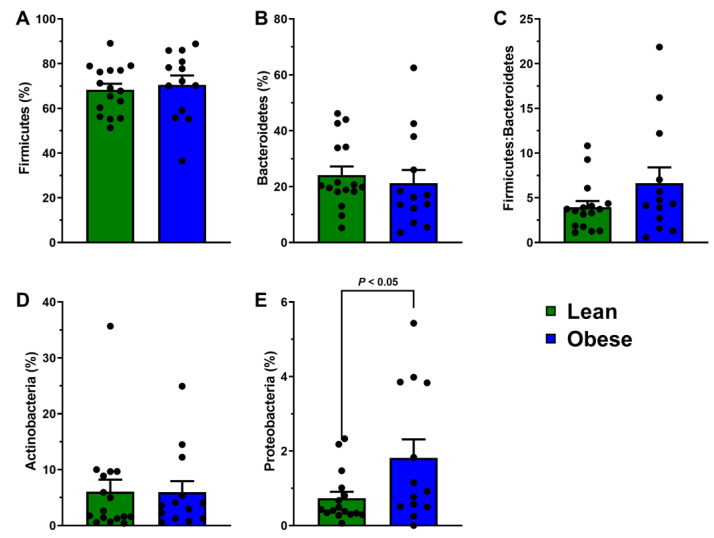
Phyla-level abundances of gut microbiota in obese and lean individuals. (**A**) Firmicutes, (**B**) Bacteroidetes, (**C**) Firmicutes:Bacteroidetes ratio, (**D**) Actinobacteria, and (**E**) Proteobacteria between obese and lean individuals. Data (means ± SEM, *n* = 13 to 16 per group) were analyzed using QIIME2 (version 2019.10).

**Figure 5:**
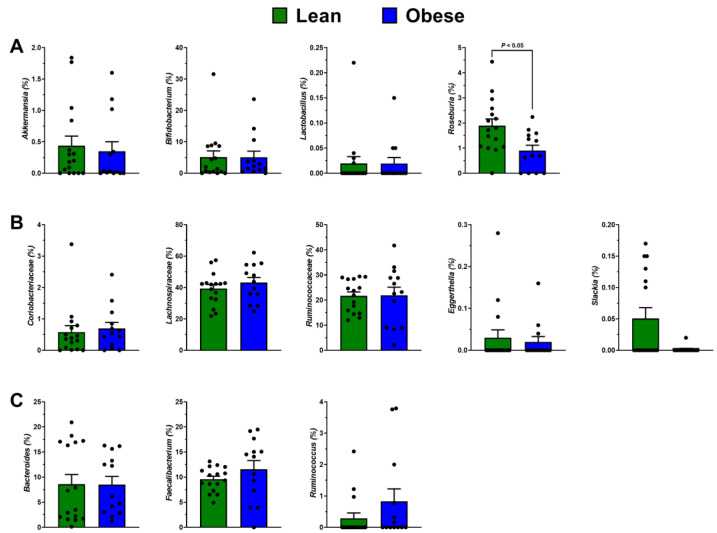
Relative abundances of family- and genus-level taxa between obese and lean individuals. Relative abundances (means ± SEM, *n* = 13 to 16 per group) of (**A**) *Akkermansia*, *Lactobacillus*, *Bifidobacterium*, and *Roseburia*, (**B**) *Coriobacteriaceae*, *Lachnospiraceae*, *Ruminococcaceae*, *Eggerthella*, and *Slackia*, and (**C**) *Bacteroides*, *Faecalibacterium*, and *Ruminococcus* between obese and lean individuals. *Slackia* was detected in too few of the participants (*n* ≤ 6 per group) to reliably conduct statistical analysis. Data were analyzed using QIIME2 (version 2019.10 obtained from http://qiime.org accessed 30 September 2022).

Because gut dysbiosis is common in obesity [15], we hypothesized that gut microbiota composition could mediate catechin and γ-VL bioavailability. Principal coordinate analysis was performed using Bray–Curtis dissimilarity to visualize group differences in microbiota composition (Figure 3A). There was no clear separation between obese and lean persons, which was corroborated by PERMANOVA that demonstrated no group-wise difference (*p* > 0.05, Figure 3A). Obese persons had significantly lower Chao1 richness compared to lean persons (Figure 3B). For Shannon index, a metric of α-diversity, a similar numerical trend was observed but this did not achieve statistical significance (*p* = 0.12; Figure 3C). At the phylum level, there was no significant difference in the relative abundances of Firmicutes, Bacteroidetes, Actinobacteria, or Firmicutes:Bacteroidetes ratio (Figure 4A–D). However, Proteobacteria abundance was greater among obese persons (Figure 4E). We also considered select genera with previously reported beneficial effects related to gut health (*Akkermansia*, *Lactobacillus*, *Bifidobacterium,* and *Roseburia*). Only *Roseburia* was depleted (*p* < 0.05) in obese persons, whereas all other genera were unaffected by obesity status (Figure 5A). We also sought to identify group-wise differences in catechin-metabolizing bacteria and bacteria associated with xenobiotic metabolism (i.e., possessing β-glucuronidase activity). The relative abundance of the families *Coriobacteriaceae, Lachnospiraceae*, and *Ruminococcaceae* and the genus *Eggerthella* were unaffected by health status. However, the genus *Slackia* was substantially lower among obese persons (Figure 5B). Lastly, the genera *Ruminococcus*, *Bacteroides*, and *Faecalibacterium*, which are known to have β-glucuronidase activity, were unaffected by obesity status (Figure 5C). 

### 3.5. Correlations between Catechin and γ-VL Pharmacokinetics, Ascorbic Acid, and Gut Microbiota Measures

**Figure 6:**
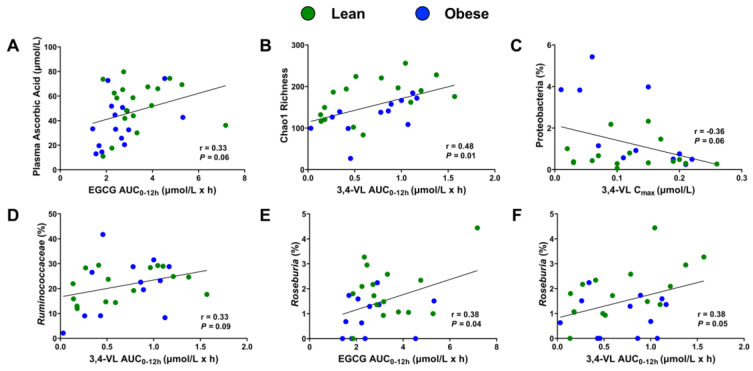
Correlations between (**A**) EGCG bioavailability and ascorbic acid, (**B**) 3,4-VL bioavailability and Chao1 richness, and (**C**–**F**) EGCG or 3,4-VL pharmacokinetic parameters with relative abundances of select taxa in lean and obese persons who completed a pharmacokinetic trial examining green tea catechin bioavailability. Pearson correlation coefficients were calculated by linear regression. Abbreviations: 3,4-VL, 5′-(3′,4′-dihydroxyphenyl)-γ-valerolactone; AUC0-12h, 12 h area under the concentration curve; C_max_, plasma maximum concentration.; EGCG, epigallocatechin gallate.

To better understand catechin and γ-VL bioavailability, we performed regression analysis between pharmacokinetic parameters and ascorbic acid, diversity metrics, and specific bacterial populations (Figure 6). Plasma ascorbic acid concentrations were positively correlated with the AUC_0-12h_ of EGC, EC, ECG (r = 0.40–0.05; *p* < 0.05) and tended to correlate with the EGCG AUC_0-12h_ (Figure 6A). EGC AUC_0-12h_ was significantly correlated with Shannon diversity index (r = 0.38; *p* < 0.05), whereas EGCG AUC_0-12h_ and C_max_ tended to positively correlate with Chao1 richness estimate and Shannon diversity index (*p* = 0.06–0.08). Plasma 3,4-VL AUC_0-12h_ was significantly positively correlated with Chao1 diversity (Figure 6B) and plasma 3,4-VL AUC_0-12h_ and plasma 3,4-VL C_max_ concentrations were also positively associated with both Chao1 diversity and Shannon diversity metrics (r = 0.38–0.48; *p* < 0.05). Plasma 3,4-VL C_max_ tended to be inversely correlated with Proteobacteria (*p* = 0.06; Figure 6C) and also with the Firmicutes:Bacteroidetes ratio (r = −0.35; *p* = 0.06). Although no parental catechins were correlated with the abundance of Bacteroidetes, the C_max_ of 3,4-VL and 3,4,5-VL were positively correlated with the relative abundance of Bacteroidetes (r = 0.41–0.51, *p* < 0.05). The AUC_0-12h_ of 3,4-VL tended to be positively associated with *Ruminococcaceae* (*p* = 0.09; Figure 6D), whereas EGCG and 3,4-VL AUC_0-12h_ were positively associated with *Roseburia* (Figure 6E,F). The ECG AUC_0-12h_ and EGCG C_max_ only showed a tendency to be positively correlated with *Roseburia* (r = 0.32–0.37, *p =* 0.05–0.09). These findings suggest that suboptimal ascorbic acid status is associated with reduced parental catechin bioavailability, while gut dysbiosis may limit γ-VL bioavailability.

## 4. Discussion

Consistent with our hypothesis, this study provides the first evidence that obesity reduces the oral bioavailability of GTE catechins. However, contrary to our hypothesis, our findings indicate that reduced catechin bioavailability in obese persons is independent of any change in microbial-derived γ-VLs in plasma and urine or the urinary elimination of catechins. Our data show that C_max_ and AUC_0-12h_, but not T_max_, were significantly reduced for all GTE catechins in obese persons. Despite the poor bioavailability of catechins, there was no difference in γ-VL bioavailability in obese persons. Consistent with no significant difference in γ-VL appearance in obesity, there were also few group-wise differences in the fecal microbiota. Indeed, obesity was only associated with reduced α-diversity, increased Proteobacteria, and lower abundances of *Roseburia* and *Slackia*. Collectively, GTE catechin bioavailability is reduced by obesity and occurs without substantially altering urinary excretion or γ-VL generation, suggesting a potential gut-level mechanism that specifically limits the absorption of GTE catechins but not their microbial metabolites.

Understanding the bioavailability of GTE catechins in both healthy and morbid populations is necessary to help establish health recommendations. The bioavailability of GTE catechins has been well studied in healthy populations [13], which is important in establishing recommendations to reduce disease risk. However, no studies have evaluated catechin bioavailability in obese populations who are at higher risk for developing chronic disease. Therefore, defining GTE catechin bioavailability will not only provide important dosing recommendations to mitigate disease but may also support health recommendations to help reverse underlying morbidity.

Differences in food matrices (gelatin-based confection vs. beverage/pill) would be expected to influence catechin bioavailability [18,41,42,43]. Regardless of health status, AUC_0-12h,_ C_max,_ and T_max_ in the present study were consistent with prior reported pharmacokinetics parameters of catechins that examined a similar dose, despite differences in catechin delivery methods [13]. Epidemiological evidence suggests that the consumption of >5 cups of brewed green tea is associated with reduced risk in cardiovascular disease-related mortality [44]. In the U.S., tea is a major source of flavonoid intake [29]. However, black tea, which is devoid of catechins, is preferred by Americans over green tea. Additionally, snacks are a growing trend to deliver bioactive food components and provide a safer alternative than GTE supplements that may lead to a higher risk of adverse events [45]. Importantly, our study showed that acute consumption of a GTE confection provided similar catechin bioavailability as GTE from a beverage or supplement. Thus, our snack-based approach may be an effective vehicle to help achieve similar consumption levels reported in epidemiological studies that suggest health benefits of fluid green tea consumption.

Metabolic complications associated with obesity (e.g., low-grade inflammation, oxidative stress, insulin resistance) are expected to influence the metabolism of dietary catechins. NFκB activation disrupts phase II and III xenobiotic metabolism [17] and could explain our findings of reduced plasma availability of GTE catechins in obesity. NFκB activation at the gut can lead to increased paracellular transport of catechins and limit their efflux back into the intestinal lumen, while at the liver, NFκB activation may inhibit phase II xenobiotic enzymes (e.g., catechol-O-methyltransferases, sulfotransferases) and also limit hepatocellular efflux of catechins by decreasing multidrug resistant protein-2 expression [17]. Endotoxin-TLR4-mediated signaling and oxidative stress are potential mechanisms by which NFκB may be activated. Consistent with this notion, obese persons in our study had an increased abundance of Proteobacteria, a major source of endotoxins. Obese persons also had significantly lower circulating ascorbic acid, which is in agreement with increased oxidative stress in obesity [46]. Because catechins can scavenge reactive oxygen/nitrogen species and/or have redox activity to assist with the recycling of oxidized ascorbic acid [47,48,49], the lower bioavailability of catechins in obese persons may reflect enhanced depletion via oxidation consistent with their purported antioxidant activity. Thus, future studies that assess catechin bioavailability in association with inflammation and oxidative stress are needed.

Our findings, as well as those from others, support that GTE catechins are poorly bioavailable with levels in plasma reaching less than 1% of the ingested dose [18]. Studies in ileostomy patients support that a majority of ingested catechins accumulate in the large intestine and are available for microbial metabolism [50]. Thus, we hypothesized that the reduced catechin bioavailability in obesity would be associated with increased microbial biotransformation of catechins to γ-VLs. Although plasma and urinary concentrations of γ-VLs (i.e., 3,4-VL, 3,4,5-VL) were consistent with prior data [23], we observed no difference in their bioavailability based on health status. Microbial metabolism, at least to 3,4-VL and 3,4,5-VL, did not account for the reduced catechin bioavailability that was observed in obese persons. Future studies that consider other microbiota-derived metabolites (e.g., upstream and downstream of γ-VLs) are necessary to provide further insight into the altered catechin bioavailability in obesity.

Importantly, our study aimed to understand the role of gut dysbiosis on catechin bioavailability and metabolism. We provide evidence herein, consistent with others, that obesity reduces α-diversity and increases Proteobacteria, suggesting the presence of gut dysbiosis [51]. The role of gut dysbiosis on catechin metabolism has yet to be determined. However, evidence from an ellagic acid bioavailability study suggests that gut dysbiosis in humans alters the production of ellagic acid-derived microbial metabolites (e.g., urolithins) [52]. Although obesity did not alter the quantity of γ-VLs, positive associations between EGCG, EGC, and 3,4-VL with α-diversity indices and inverse associations with Proteobacteria abundance and Firmicutes:Bacteroidetes ratio suggest that greater bioavailability of catechins and γ-VLs occurs with reduced gut dysbiosis. Indeed, increases in the Bacteroidetes phylum have been associated with leaner individuals, weight loss in obese persons, and increased functional diversity [53,54]. Notably, the C_max_ for 3,4-VL and 3,4,5-VL were positively associated with increases in Bacteroidetes, which have shown to possess deglycosylation capabilities [24]. However, catechins are not present as glycosides. Thus, other members of the Bacteroidetes phyla may potentially possess catechin-metabolizing functionalities that promote γ-VL production. 

We also considered that gut dysbiosis may influence the abundance in catechin-metabolizing bacteria and bacteria with xenobiotic capabilities that may influence catechin bioavailability in obesity. The families *Coriobacteriaceae*, *Lachnospiraceae*, and *Ruminococcaceae* and the genera *Eggerthella* and *Slackia* have been identified as having specific flavonoid metabolizing activities in vitro (i.e., C-ring cleavage, dehydroxylation) [24]. We show that only the abundance of *Slackia* was reduced in obesity, but it was not correlated with γ-VL bioavailability. Interestingly, *Slackia* was only detected in one obese participant from the present cohort consistent with its low prevalence during obesity [55], while it was detected in six lean persons. Although *Slackia* represents only a small percentage of the total gut bacteria, its presence or absence along with its C-ring cleavage activities [56] could lead to the production of certain microbial metabolites that are observed in a small proportion of participants (e.g., 3,4,5-VL) and may drive inter-individual differences [57]. In addition, we considered certain bacterial genera (*Ruminococcus*, *Bacteroides*, *Faecalibacterium*, and *Roseburia*) that are known to possess β-glucuronidase activity [58,59] that may influence catechin bioavailability. Catechins and microbial metabolites undergo phase II metabolism in the liver or small intestine to form glucuronidated metabolites [60]. Glucuronidated metabolites may accumulate in the gut lumen through intestinal efflux or excretion in the bile [61], deconjugated by bacterial β-glucuronidases and reabsorbed or further metabolized by the gut microbiota to γ-VLs. *Roseburia* abundance was significantly lower in obese persons and was positively associated with EGCG and 3,4-VL bioavailability, suggesting increased bacterial genera with β-glucuronidase activity may substantially potentiate catechin/γ-VL bioavailability. However, our study was limited to analyzing deconjugated catechins and γ-VLs; thus, future studies that assess conjugated and unconjugated catechins in association with the gut microbiota are warranted.

## 5. Conclusions

Overall, our findings indicate that obesity reduces GTE catechin bioavailability that occurs independent of γ-VL accumulation and relative abundances of catechin-metabolizing bacteria. These findings suggest that obese persons require higher intakes of catechins to achieve steady state concentrations to match those of lean individuals in order to realize the cardiometabolic benefits of green tea catechins. Alternatively, if γ-VLs are the health beneficial metabolites in humans, obese individuals would not require higher catechin intakes. Therefore, future studies investigating the independent functional activities of catechins and γ-VLs relative to cardiometabolic health outcomes are critically needed. In addition, our analytical approach only measured total (i.e., free and deconjugated) catechins/γ-VLs. This may limit an understanding of phase II catechin metabolite bioavailability in obesity. Differences in phase II metabolites may occur due to disease alterations [62], polymorphisms [63], and gut microbiota functions [16] and are important to consider, especially since phase II catechin metabolites may possess biological activities [64]. Additionally, participants consumed the GTE confection after an overnight fast. However, prior studies have indicated that catechin bioavailability can be altered by their ingestion in the fasted state or with food [65]. Since our goal is to incorporate GTE catechins into the American diet, studies are needed to establish whether food/confection interactions alter catechin bioavailability. Lastly, obese persons in this study were relatively metabolically healthy, despite greater BMI, waist circumferences, and insulin resistance. They had elevated blood pressure and glucose, as well as lower HDL, but these values were within normal clinical limits. The relative healthiness of the obese individuals may explain the lack of significant changes in γ-VL bioavailability and specific gut microbiota abundances. In conclusion, our study provides a framework for defining precise GTE catechin doses in obese populations for future targeted clinical interventions focused on assessing the antioxidative and anti-inflammatory benefits of GTE catechins.

## Figures and Tables

**Table 1:** Characteristics of lean and obese persons who completed a pharmacokinetics trial.

	Lean	Obese	*p* Value
Gender	10 M:9 F	10 M:7 F	
Age (y)	27.1 ± 1.7	30.7 ± 2.0	0.18
BMI (kg/m^2^)	21.8 ± 0.39	33.5 ± 0.72	*0.0001*
Waist Circumference (cm)	76.2 ± 1.3	104.4 ± 2.9	*0.0001*
Systolic BP (mmHg)	112.1 ± 2.3	124.4 ± 2.3	*0.0006*
Diastolic BP (mmHg)	72.5 ± 1.6	81.0 ± 1.6	*0.0006*
Plasma Glucose (mg/dL)	92.3 ± 1.8	95.7 ± 2.1	0.24
Plasma Triglyceride (mg/dL)	61.7 ± 7.5	74.7 ± 8.0	0.25
Plasma Total Cholesterol (mg/dL)	181.1 ± 7.8	172.2 ± 7.8	0.43
Plasma HDL (mg/dL)	53.6 ± 2.8	43.0 ± 2.8	*0.012*
Insulin (uIU/mL)	7.6 ± 0.9	12.3 ± 1.4	*0.007*
HOMA-IR	1.7 ± 0.2	2.9 ± 0.3	*0.004*
ALT (U/L)	15.6 ± 1.0	19.1 ± 1.6	*0.07*
AST (U/L)	16.3 ± 0.4	17.4 ± 0.9	0.25
Plasma Ascorbic Acid (µmol/L)	53.2 ± 4.6	37.3 ± 4.7	*0.023*
Plasma Uric Acid (µmol/L)	318.6 ± 14.2	347.1 ± 14.4	0.17

Data are means ± SEM. Abbreviations: ALT, alanine aminotransferase; AST, aspartate aminotransferase; BP, blood pressure; BMI, body mass index; HDL, high-density lipoproteins.

**Table 2:** Pharmacokinetic parameters of plasma catechins.

	EGCG		EGC		EC		ECG	
	Lean	Obese	Lean	Obese	Lean	Obese	Lean	Obese
AUC_0-12h_	3.46 ± 0.3	2.62 ± 0.3 *	1.18 ± 0.1	0.88 ± 0.1 *	0.78 ± 0.1	0.57 ± 0.04 *	1.06 ± 0.1	0.78 ± 0.1 *
(µmol/L × h)								
AUC_0-12h_	39.2%	39.3%	29.2%	31.0%	28.5%	24.7%	37.7%	38.7%
(CV_Inter_)								
C_max_	0.50 ± 0.04	0.38 ± 0.03 *	0.22 ± 0.01	0.18 ± 0.01 *	0.16 ± 0.01	0.13 ± 0.01 *	0.14 ± 0.01	0.09 ± 0.01 *
(µmol/L)								
T_max_ (h)	3.32 ± 0.25	2.65 ± 0.26	1.92 ± 0.25	1.41 ± 0.15	1.66 ± 0.24	1.53 ± 0.19	4.05 ± 0.24	3.94 ± 0.39
12 h Plasma Concentration (µmol/L)	0.13 ± 0.01	0.11 ± 0.01	0.02 ± 0.003	0.02 ± 0.003	0.01 ± 0.002	0.01 ± 0.002	0.06 ± 0.01	0.05 ± 0.01

Data are means ± SEM, *n* = 17–19 per group. * indicates statistical difference between lean and obese persons (*p* < 0.05). Abbreviations: AUC_0-12h_, 12 h area under the concentration curve, C_12h_, plasma concentration at 12 h; C_max_, plasma maximum concentration; CV_Inter_, interindividual coefficients of variation; EC, epicatechin; ECG, epicatechin gallate; EGC, epigallocatechin; EGCG, epigallocatechin gallate; T_max_, time to reach maximum plasma concentration.

**Table 3:** Pharmacokinetic parameters of plasma γ-VLs.

	3,4-VL		3,4,5-VL	
	Lean	Obese	Lean	Obese
# participants detected (%)	19/19 (100%)	17/17 (100%)	14/19 (74%)	13/17 (76%)
AUC_0-12h_ (µmol/L × h)	0.70 ± 0.12	0.74 ± 0.12	0.18 ± 0.06	0.19 ± 0.03
AUC_0-12h_ (CV_Inter_)	66.1%	59.0%	125.8%	61.9%
C_max_ (µmol/L)	0.12 ± 0.02	0.13 ± 0.02	0.041 ± 0.01	0.043 ± 0.01
T_max_ (h)	9.1 ± 0.56	7.94 ± 0.72	9.07 ± 0.74	8.92 ± 0.66

Data are means ± SEM. Abbreviations: 3,4-VL, 5′-(3′,4′-dihydroxyphenyl)-γ-valerolactone; 3,4,5-VL, 5′-(3′,4′,5′-trihydroxyphenyl)-γ-valerolactone; AUC_0-12h_, 12 h area under the concentration curve; C_max_, plasma maximum concentration; CV_Inter_, interindividual coefficients of variation; T_max_, time to reach maximum plasma concentration.

**Table 4:** Twenty-Four Hour urinary accumulation of parental catechins and γ-VLs.

	Total_0-24h_ (mg Excreted)		% Dose Recovered ^a^	
	Lean	Obese	Lean	Obese
EGCG	0.03 ± 0.01	0.02 ± 0.003	0.01 ± 0.002	0.01 ± 0.001
EGC	1.32 ± 0.13	1.09 ± 0.09	1.51 ± 0.15	1.25 ± 0.10
EC	0.72 ± 0.07	0.60 ± 0.05	1.84 ± 0.19	1.55 ± 0.12
ECG	0.003 ± 0.001	0.002 ± 0.001	0.01 ± 0.004	0.01 ± 0.003
3,4-VL	3.32 ± 0.49	3.27 ± 0.46	0.75 ± 0.11	0.74 ± 0.10
3,4,5-VL	0.88 ± 0.27	0.91 ± 0.19	0.23 ± 0.07	0.24 ± 0.05

Data are means ± SEM. Abbreviations: 3,4-VL, 5′-(3′,4′-dihydroxyphenyl)-γ-valerolactone; 3,4,5-VL, 5′-(3′,4′,5′-trihydroxyphenyl)-γ-valerolactone; EC, epicatechin; ECG, epicatechin gallate; EGC, epigallocatechin; EGCG, epigallocatechin gallate. ^a^ Dose recovered was calculated by dividing the quantity of mg excreted of each catechin and divided by the amount in the initial dose. 3,4-VL % dose recovered was calculated from mg excreted 3,4-VL divided by the sum of all catechins in dose. 3,4,5-VL % dose recovered was calculated by dividing mg excreted of 3,4,5-VL from the sum of EGCG and EGC in dose.

## Data Availability

Not applicable.
